# Study on the Targeted Improvement Mechanism of the Carrier Concentration and Mobility of BiCuSeO Ceramics

**DOI:** 10.3390/mi14091757

**Published:** 2023-09-10

**Authors:** Zhibin Wang, Hong Zhao, Xinyu Luo, Wenyuan Han, Hao Wang, Linghao Meng, Xinqi She, Anlong Quan, Yixin Peng, Guoji Cai, Yi Liu, Yong Tang, Bo Feng

**Affiliations:** 1 Institute of Engineering and Technology, Hubei University of Science and Technology, Xianning 437100, China17710522492@163.com (W.H.); qal205347618@163.com (A.Q.); 15271899946@163.com (Y.P.); 2 School of Mechanical and Electrical Engineering, Wuhan Donghu University, Wuhan 430070, China; 3 The State Key Laboratory of Refractories and Metallurgy, Wuhan University of Science and Technology, Wuhan 430081, China

**Keywords:** carrier concentration, carrier mobility, Al doping, functional ceramics

## Abstract

BiCuSeO has great application prospects in thermoelectric power generation and thermoelectric catalysis, but it is limited by its lower thermoelectric performance. Herein, BiCuSeO bulk materials were prepared using a solid-phase reaction method and a ball-milling method combined with spark plasma sintering, and then the thermoelectric properties were improved by synergistically increasing carrier concentration and mobility. Al was adopted to dope into the BiCuSeO matrix, aiming to adjust the carrier mobility through energy band adjustment. The results show that Al doping would widen the bandgap and enhance the carrier mobility of BiCuSeO. After Al doping, the thermoelectric properties of the material are improved in the middle- and high-temperature range. Based on Al doping, Pb is adopted as the doping element to dope BiCuSeO to modify the carrier concentration. The results show that Al/Pb dual doping in the BiCuSeO matrix can increase the carrier concentration under the premise of increasing carrier mobility. Therefore, the electrical conductivity of BiCuSeO can be improved while maintaining a large Seebeck coefficient. The power factor of Al/Pb doping reached ~7.67 μWcm^−1^K^−2^ at 873 K. At the same time, the thermal conductivity of all doped samples within the test temperature range maintained a low level (<1.2 Wm^−1^K^−1^). Finally, the *ZT* value of the Al/Pb-doped BiCuSeO reached ~1.14 at 873 K, which is ~2.72 times that of the pure phase, and the thermoelectric properties of the matrix were effectively improved.

## 1. Introduction

As the energy crisis emerges, waste heat recovery has become one of the effective ways to solve the problem of energy shortage [1]. Thermoelectric materials play a pivotal role in the waste-heat-collection process. Thermoelectric materials have broad application prospects in fields such as isotope batteries for aerospace, high-temperature flue-gas-waste-heat recovery power generation, high-temperature steel billet waste-heat-power generation, and geothermal power generation. There are over 200 types, including low-temperature thermoelectric materials, medium-temperature thermoelectric materials, high-temperature thermoelectric materials, etc. Among them, oxygenated thermoelectric materials have strong oxidation resistance, good thermal stability, low cost, and a simple preparation process; they are also non-toxic, non-polluting, and have a long service life and other advantages compared with traditional alloy-based materials, but due to low electrical conductivity, the performance in oxygenated thermoelectric materials is limited [2]. A large number of studies have found that the thermoelectric properties of oxygenated thermoelectric materials can be effectively improved through donor/acceptor effect doping, and oxide thermoelectric materials have once again attracted the attention of researchers [3].

BiCuSeO is a layered compound consisting of an insulating layer (Bi_2_O_2_)^2+^ and a conductive layer (Cu_2_Se_2_)^2−^ overlapping each other. BiCuSeO exhibits a large Seebeck coefficient (~380 μVK^−1^) at room temperature, relatively low thermal conductivity (~0.9 Wm^−1^K^−1^), and low electrical conductivity (~1 Scm^−1^) [4]. According to the *ZT* value calculation formula (*ZT* = *σS*^2^*T*/*κ*, *σ* is the electrical conductivity, *S* is the Seebeck coefficient, and *κ* is the thermal conductivity) [5], for such a material with low thermal conductivity, the *ZT* value can be increased by increasing the material’s *σ* and *S*, or by reducing the *κ*. Focusing on at the problem of low *σ*, a large number of studies have adopted positive monovalent elements (such as Na [6]/Ag [7]/Cs [8], etc.) or positive bivalent elements (such as Mg [9]/Ca [10]/Sr [11]/Ba [12]/Pb [13,14], Ca-Pb dual doping [15], Pb-Te dual doping [16], Pb-Se dual doping [17], etc.) doped at Bi sites to introduce acceptor effects. The strategy could increase the carrier concentration and *σ* but greatly reduce the mobility and increase the carrier thermal conductivity, which would be harmful to the improvement of *ZT*.

This work aimed to optimize thermoelectric performance by simultaneously optimizing carrier concentration and mobility through dual position doping. Al, selected through first-principle calculation, was used to dope at the Cu site to adjust the energy band structure, improve the mobility, and initially adjust the thermoelectric properties. The raw material of Al is very cheap and non-toxic, which is conducive to the advancement of the industrial application of BiCuSeO. The effect of Al doping on thermoelectric performance was explored through a combination of simulation and experimental testing on the basis of Al doped at Cu sites, combined with Pb doped at Bi sites for double doping, in order to achieve the effect of synergistic improvement of carrier concentration and mobility and enhance the thermoelectric conversion efficiency.

## 2. Experimental Part

The corresponding concentration of BiCu_1−*x*_Al*_x_*SeO and Bi_1−*x*_Pb*_x_*Cu_1−*x*_Al*_x_*SeO (*x* = 0, 0.01, 0.02, 0.03, 0.04, 0.05) is weighed according to the stoichiometric ratio, and the material is uniformly mixed through a ball mill. Then, the mixed materials are placed in a glass test tube, and the air in the glass test tube is extracted by a vacuum instrument to keep the vacuum degree in the tube at 5 × 10^−2^ Pa, and the tube is sealed. The sealed tube is placed in a muffle furnace for solid-phase reaction to synthesize the corresponding structure. The bulk samples of BiCu_1−*x*_Al*_x_*SeO and Bi_1−*x*_Pb*_x_*Cu_1−*x*_Al*_x_*SeO (*x* = 0, 0.01, 0.02, 0.03, 0.04, 0.05) are prepared using a graphite indenter to directly a apply pulsed current to the particles in a cylindrical graphite mold for rapid SPS sintering. The sintering pressure is 60 MPa, and a 3 min uniform pressure increase and a 3 min uniform pressure decrease method is used. The heating rate during the sintering process is 50 K per minute, and the cooling rate is 50 K per minute. The circulating water is cooled by a condensing tower. The time for holding and pressing during the sintering process is 15 min.

After preparation of thermoelectric materials is completed, the BiCu_1−*x*_Al*_x_*SeO and Bi_1−*x*_Pb*_x_*Cu_1−*x*_Al*_x_*SeO (*x* = 0, 0.01, 0.02, 0.03, 0.04, 0.05) samples need to be characterized in terms of structure, morphology, and thermoelectric properties. At the same time, based on first-principle calculations, we explored the influence of Al doping on the electronic structure of BiCuSeO from the electronic energy band and density of states. Characterization methods in terms of structure and morphology are as follows: (1) X-ray diffraction (XRD) to study the crystal structure of the prepared sample material: the X-ray diffractometer (XRD, DX-2700, Dandong Fangyuan Instrument Co., Ltd., Liaoning, China) is used to analyze the phase structure of the sample. XRD uses Cu-Kα as the ray source, the wavelength is 0.15406 nm, the rotating anode, the step length of the instrument during the test is 0.03°, and the working current is 40 mA; (2) Scanning electron microscope (FESEM) to study the internal morphology of the prepared sample: in order to directly observe the morphology of the prepared sample at the micron or nanometer level, a scanning electron microscope produced by a Japanese company (FESEM, JSM-7001F, JEOL Co., Ltd., Tokyo, Japan) is used to observe the fracture morphology of the samples, and use its own energy spectrometer (EDS) to analyze the element distribution of the fracture morphology of the sample. Characterization methods in terms of thermoelectric performance are as follows: (1) The tests of carrier concentration and carrier mobility of the sample in the experiment all use the DC Hall test system (ET9005, East Changing Technologies, Inc., Washington, DC, USA); (2) The experimental electrical performance tests are all conducted using a static DC thermoelectric test system (ZEM-3, ULVAC-RIKO, Inc., Yokohama, Japan), and the samples of Bi_1−*x*_Pb*_x_*Cu_1−*x*_Al*_x_*SeO (*x* = 0, 0.01, 0.02, 0.03, 0.04, 0.05) are protected in a low-pressure helium (He) environment during the test; (3) The thermal performance of the experimental sample is tested using a laser thermal conductivity meter (LFA457), and the sample is tested under argon (Ar) protection.

## 3. Experimental Test Results and Analysis

**Figure 1:**
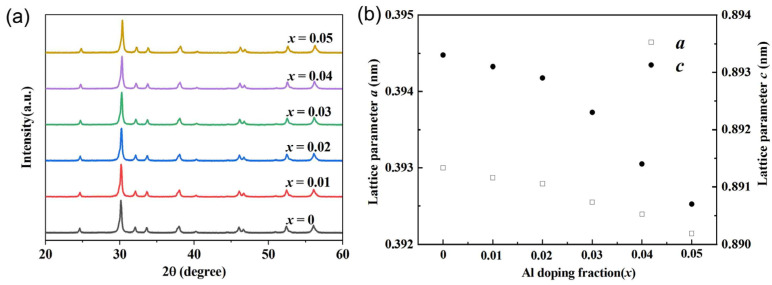
(**a**) Comparison of X-ray diffraction peaks of BiCu_1−*x*_Al*_x_*SeO (*x* = 0, 0.01, 0.02, 0.03, 0.04, 0.05) samples; (**b**) corresponding lattice constant of BiCu_1−*x*_Al*_x_*SeO (*x* = 0, 0.01, 0.02, 0.03, 0.04, 0.05) samples.

**Figure 2:**
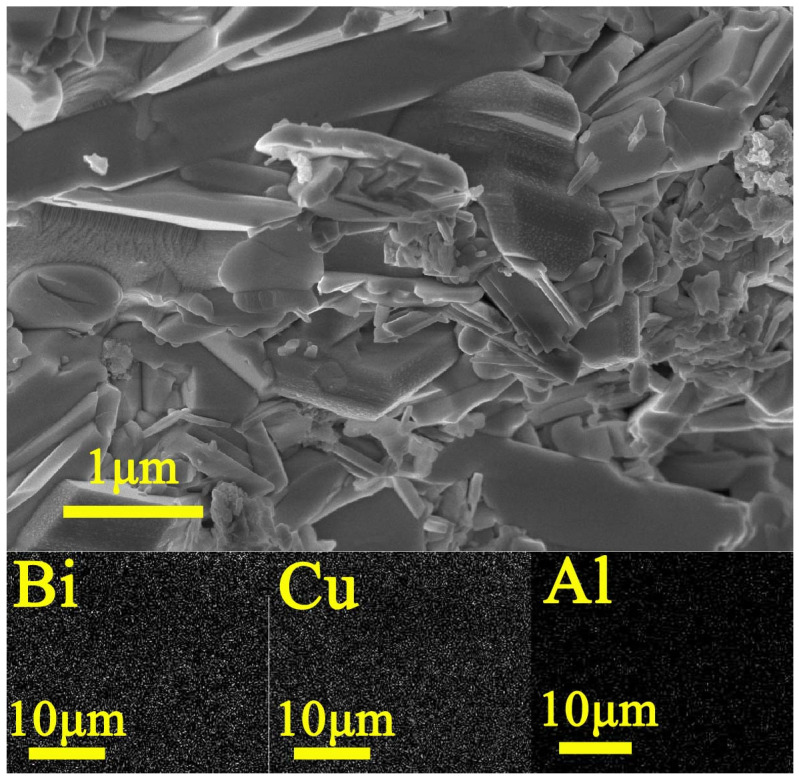
The scanning electron micrograph (SEM) of the cross-section and the energy spectrum of the polished surface of the sample with *x* = 0.05 for the BiCu_1−*x*_Al*_x_*SeO (*x* = 0, 0.01, 0.02, 0.03, 0.04, 0.05) samples.

It can be seen from Figure 1a that all XRD diffraction peaks of BiCu_1−*x*_Al*_x_*SeO (*x* = 0, 0.01, 0.02, 0.03, 0.04, 0.05) are consistent with the ZrSiCuAs structure, and no second phase formation is observed in all samples. It is worth noting that the lattice constant after Al doping as shown in Figure 1b becomes smaller than that of the undoped. The reason for this phenomenon can be attributed to the difference in ionic radius between the doping element Al (~0.0535 nm) and the substituted element Cu (~0.077 nm) for Vegard’s law [18]. It can be clearly seen from Figure 2 that the grain size of the sample is about ~1–5 μm. The sample exhibits good crystallinity, the grains of the sample are tightly stacked, and the surface has no holes. At the same time, we used an energy spectrometer (EDS) to analyze the energy spectrum of the polished surface of the sample with *x* = 0.05. It can also be found that the distribution of various elements in the sample is relatively uniform. No aggregation or segregation of elements occurred.

**Figure 3:**
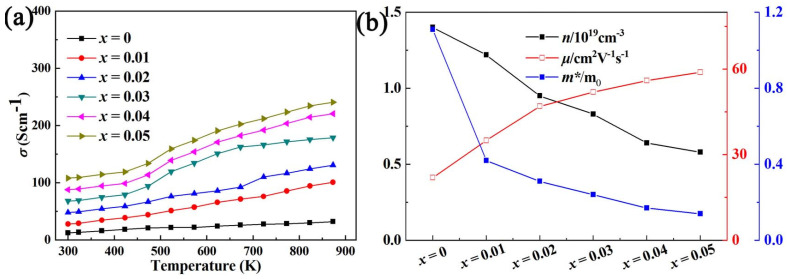
(**a**) The relationship between electrical transport of BiCu_1−*x*_Al*_x_*SeO (*x* = 0, 0.01, 0.02, 0.03, 0.04, 0.05) with temperature; (**b**) the relationship between ***n***, ***μ***, m* of BiCu_1−*x*_Al*_x_*SeO (*x* = 0, 0.01, 0.02, 0.03, 0.04, 0.05) with doping content.

**Figure 4:**
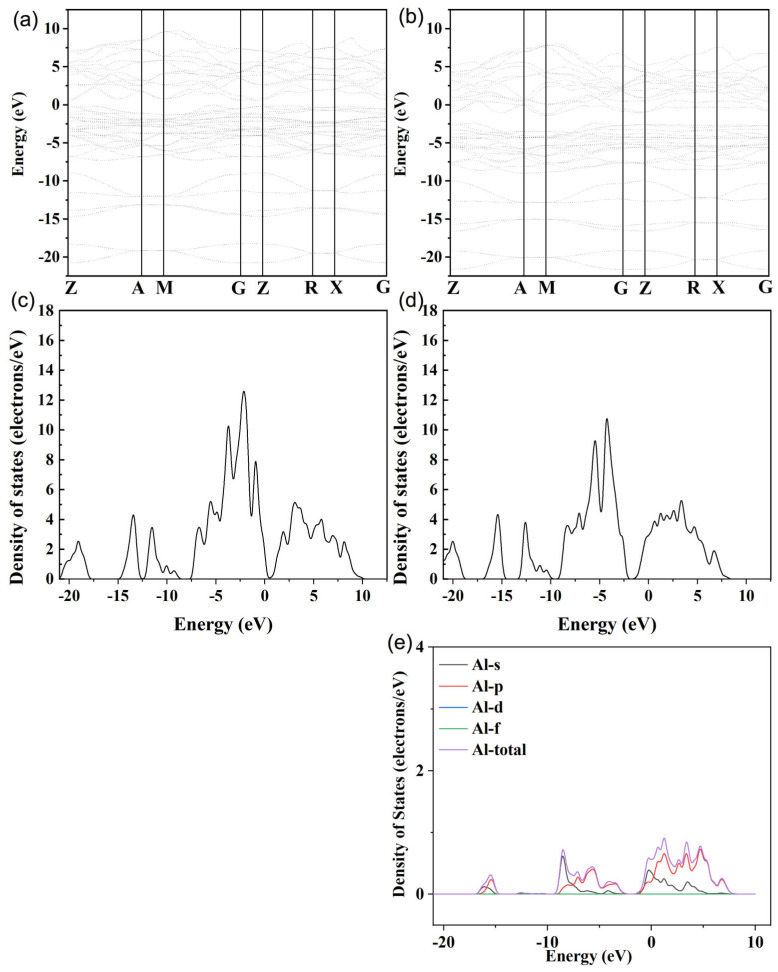
The calculated energy band and density of states by CASTEP of materials studio ((**a**,**c**) for the undoped, (**b**,**d**) for Al-doped, (**e**) for the PDOS of Al).

**Figure 5:**
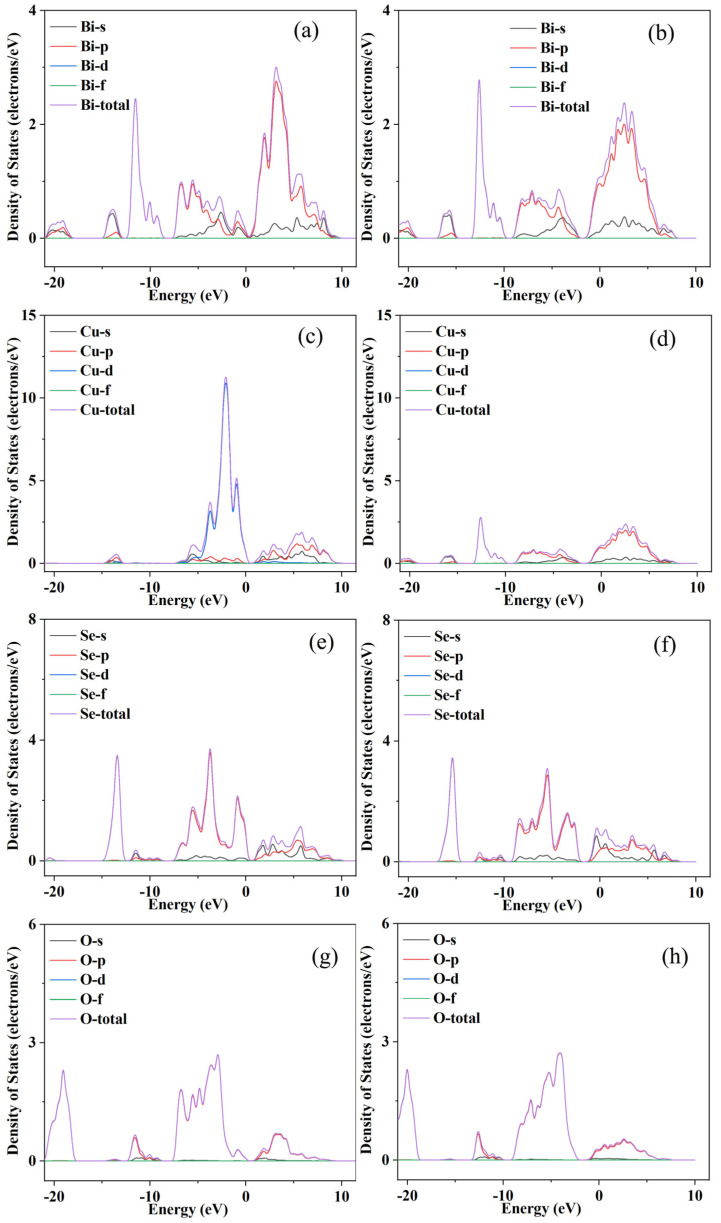
The calculated PDOS ((**a**,**c**,**e**,**g**) for the undoped and (**b**,**d**,**f**,**h**) for Al-doped).

It can be clearly seen from Figure 3a that Al element doping can significantly increase the electrical conductivity (*σ*) of BiCuSeO, and with the increase in the doping concentration, *σ* becomes continuously larger. The *σ* obtained at all doping concentrations increases with the increase in temperature in the range of 300–873 K. At 873 K, the best *σ* is obtained at a concentration of *x* = 0.05, and its value is ~240.73 Scm^−1^, which is about nine times the value of the pure phase at the same temperature. At the same time, in order to more directly grasp the inherent reason for the increase in *σ* after Al doping, we conducted a Hall test at room temperature on the BiCu_1−*x*_Al*_x_*SeO (*x* = 0, 0.01, 0.02, 0.03, 0.04, 0.05) samples. Figure 3b shows the carrier concentration and carrier mobility of samples with different doping concentrations at room temperature. The carrier concentration of the samples decreased after Al element doping, but its carrier mobility increased from ~22.01 cm^2^V^−1^s^−1^ to ~58.32 cm^2^V^−1^s^−1^ when the doping concentration was 5%, which indicates that Al element doping can increase the carrier mobility of the material, thereby increasing its *σ*. Room-temperature Hall test results show that Al doping can widen the bandgap and enhance the carrier mobility of the material. After Al doping, the thermoelectric properties of the material are improved in the middle- and high-temperature range. The increase in bandgap caused a lower carrier concentration and then carrier mobility. Furthermore, Al is a lighter element compared to Bi, which may be the other reason why Al doping could improve the carrier mobility. This is different from alkali metal or alkaline earth metal doping, which increases *σ* by increasing the carrier concentration of the material [9,10,11,12]. The results of the energy band calculation from the materials studio as shown in Figure 4 and Figure 5 confirmed the testing results. The valence band and conduction band moves down, the partial density of states (PDOS) near Fermi level for Bi/Cu/Se/O atoms decreases, and the bandgap becomes wider after Al doping. This would lead to two results. On the one hand, it is more difficult for carriers to jump over the forbidden band, and the carrier concentration decreases [19]. On the other hand, the widening of the bandgap would make the capacity difference between the heavy band and the light band smaller, and the increase in the light band’s contribution would increase the mobility [20].

**Figure 6:**
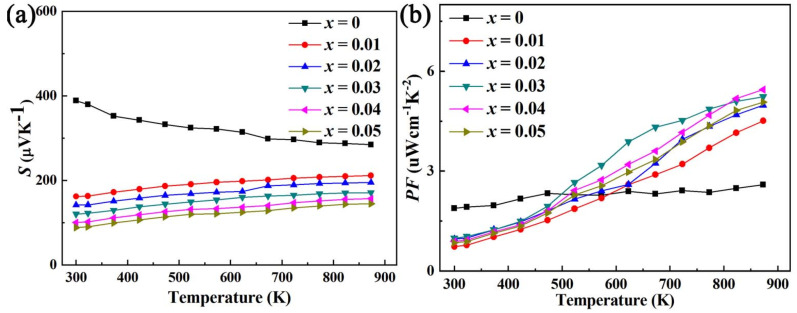
(**a**) The relationship between Seebeck coefficient (*S*) and (**b**) power factor (*PF*) of BiCu_1−*x*_Al*_x_*SeO (*x* = 0, 0.01, 0.02, 0.03, 0.04, 0.05) with temperature.

As shown in Figure 6, the Seebeck coefficients (*S*) of BiCu_1−*x*_Al*_x_*SeO (*x* = 0, 0.01, 0.02, 0.03, 0.04, 0.05) samples are positive, which indicates that all samples exhibit a p-type semiconductor characteristic. *S* after Al doping is still relatively large, and the Seebeck coefficient at 873 K remains at ~146.231 μV/K–~211.398 μV/K. *PF* of all the Bi_1−*x*_Pb*_x_*Cu_1−*x*_Al*_x_*SeO (*x* = 0, 0.01, 0.02, 0.03, 0.04, 0.05) samples increases with increasing temperature. The *PF* (power factor) of the sample doped with the Al element obtains the maximum value at *x* = 0.02, and *PF* reaches the maximum value of ~5.45 μWcm^−1^K^−2^ at 873 K.

**Figure 7:**
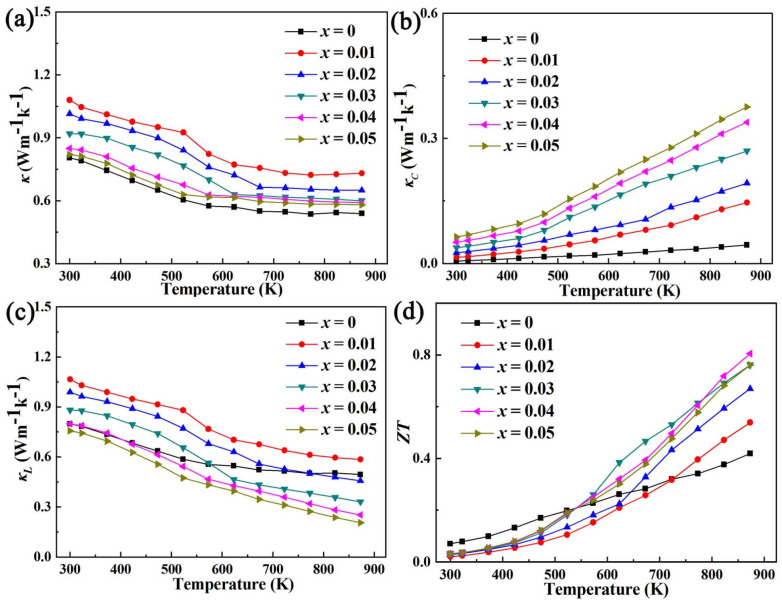
The relationship between thermal transport and *ZT* of BiCu_1−*x*_Al*_x_*SeO (*x* = 0, 0.01, 0.02, 0.03, 0.04, 0.05) with temperature.

As shown in Figure 7a, the thermal conductivity (*κ*) of all the BiCu_1−*x*_Al*_x_*SeO (*x* = 0, 0.01, 0.02, 0.03, 0.04, 0.05) samples is relatively small compared with other oxide thermoelectric materials [21]. With the increase in temperature, the thermal conductivity of the BiCu_1−*x*_Al*_x_*SeO (*x* = 0, 0.01, 0.02, 0.03, 0.04, 0.05) sample remains below ~1.2 Wm^−1^K^−1^. After Al doping, the thermal conductivity shows a trend in which it first increases and then decreases. When the doping amount is low, the thermal conductivity increases. On the one hand, the carrier thermal conductivity (*κ_C_*) as shown in Figure 7b increases with the increase in electrical conductivity [22]. On the other hand, Al (26.98) is a “light” element, relative to Cu (63.55). The substitution of Al for Cu would cause a reduction in heavy elements’ content, which would increase the lattice thermal conductivity (*κ_L_*) as shown in Figure 7c [23]. When the doping amount increases, the replacement of Cu by Al would cause a change in the point defects and lattice strain field, thereby increasing phonon scattering [24,25]. When the effect is stronger than the influence of light elements, it would cause a decrease in lattice thermal conductivity and total thermal conductivity.

Figure 7d shows the relationship between the *ZT* value of different Al element doping concentrations and the temperature within the test temperature range. From the graph of the change of *ZT* with temperature, it can be seen that all *ZT* values increase with the increase in temperature for the BiCu_1−*x*_Al*_x_*SeO (*x* = 0, 0.01, 0.02, 0.03, 0.04, 0.05) samples. In the low-temperature region, the *ZT* values of the BiCu_1−*x*_Al*_x_*SeO (*x* = 0, 0.01, 0.02, 0.03, 0.04, 0.05) samples decreases due to the decrease in the power factor, but in the medium- and high-temperature range, the *ZT* value increases significantly. The *ZT* value reaches the maximum under the doping concentration *x* = 0.04, which is ~0.81 at 873 K, which is about ~92% higher than that of the pure phase.

**Figure 8:**
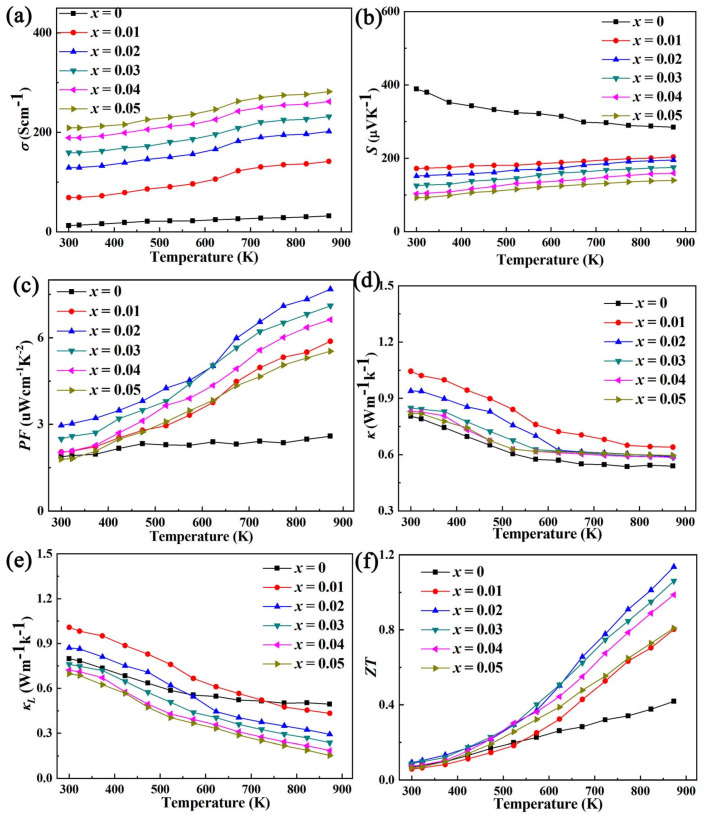
The relationship between electrical/thermal transport and *ZT* of Bi_1−*x*_Pb*_x_*Cu_1−*x*_Al *_x_*SeO (*x* = 0, 0.01, 0.02, 0.03, 0.04, 0.05) with temperature.

**Figure 9:**
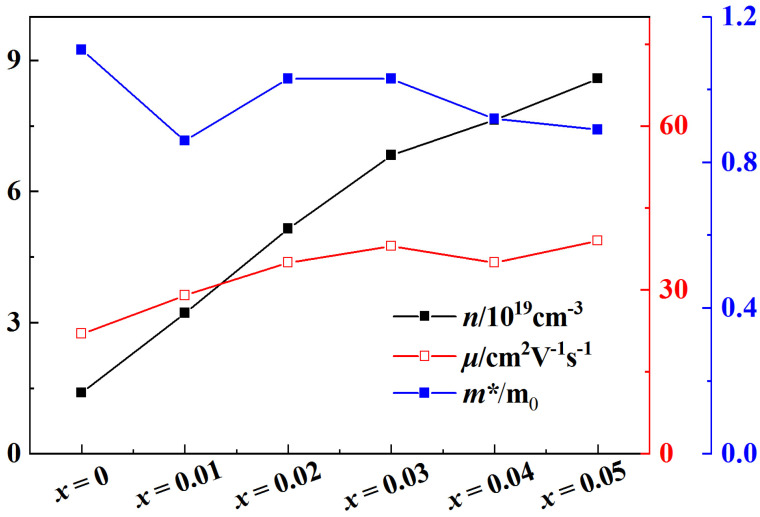
The relationship between the carrier concentration (*n*), mobility (*μ*), effective mass (m^*^), and *ZT* of Bi_1−*x*_Pb*_x_*Cu_1−*x*_Al *_x_*SeO (*x* = 0, 0.01, 0.02, 0.03, 0.04, 0.05).

In view of the significant effect of Al doping on improving mobility, Pb doping is used to increase carrier concentration [14], combined with Al doping to increase mobility for double doping as shown in Figure 8. It can be seen that the effect of Pb/Al double doping to improve the electrical conductivity and *ZT* value is more obvious than that of Al doping for that of Pb, as a +2 valence element, doping at the Bi site (+3 valence) of BiCuSeO would introduce holes, which is the acceptor effect, thereby increasing the carrier concentration [13,26,27,28]. The best *σ* of the Bi_1−*x*_Pb*_x_*Cu_1−*x*_Al*_x_*SeO (*x* = 0, 0.01, 0.02, 0.03, 0.04, 0.05) samples is obtained at a concentration of *x* = 0.05, and its value is ~282.59 Scm^−1^. The reason for this is that Pb/Al doping can contribute to the increase in carrier concentration and mobility as shown in Figure 9. The *ZT* value reaches the maximum under the doping concentration *x* = 0.02, which is ~1.14 at 873 K, which is ~2.72 times that of the pure phase.

## 4. Conclusions

In summary, pristine BiCuSeO bulk materials were prepared by the solid-phase reaction method and ball-milling method combined with spark plasma sintering, and then the thermoelectric properties were improved by doping Al and Pb. Al was adopted to dope into the BiCuSeO matrix, hoping to adjust the carrier mobility through its energy band adjustment and realize the improvement in thermoelectric performance. Room-temperature Hall test results show that Al doping can widen the bandgap and enhance the carrier mobility of the material. After Al doping, the thermoelectric properties of the material are improved in the middle- and high-temperature range. Based on Al doping, Pb is used as the doping source to dope BiCuSeO to modify the carrier concentration. The results show that Al/Pb dual doping in the BiCuSeO matrix can increase the carrier concentration and the carrier mobility. Therefore, the electrical conductivity of the material can be improved while maintaining a large Seebeck coefficient. The power factor of Al/Pb doping can reach 7.67 μWcm^−1^K^−2^ at 873 K. At the same time, the thermal conductivity of all doped samples within the test temperature range maintains a low value (<1.2 Wm^−1^K^−1^). Finally, the *ZT* value of Al/Pb-doped BiCuSeO reaches ~1.14 at 873 K, which is ~2.72 times that of the pure phase, and the thermoelectric properties of the matrix are effectively improved. Next, structural optimization based on the current composition optimization is planned to be carried out, as well as a combination of the two to improve thermoelectric performance.

## Data Availability

The raw/processed data required to reproduce these findings cannot be shared at this time as the data also form part of an ongoing study.
